# Effect of vitamin D supplementation on physical performance and activity in non-western immigrants

**DOI:** 10.1530/EC-14-0096

**Published:** 2014-11-21

**Authors:** Mirjam M Oosterwerff, Rosa Meijnen, Natasja M Van Schoor, Dirk L Knol, Mark H H Kramer, Mireille N M Van Poppel, Paul Lips, E Marelise W Eekhoff

**Affiliations:** 1 Department of Internal Medicine, Endocrine Section; 2 Epidemiology and Biostatistics, EMGO Institute for Health and Care Research; 3 Public and Occupational Health, EMGO Institute for Health and Care Research, VU University Medical Center, P.O. Box 7057, Amsterdam, 1007 MB, The Netherlands

**Keywords:** randomized controlled trial, vitamin D supplementation, overweight, non-western immigrants, 6-min walk test, physical activity questionnaire, accelerometry, physical activity, physical performance

## Abstract

Vitamin D deficiency is highly prevalent among non-western immigrants in The Netherlands and associated with poor physical performance. The aim of this study was to assess the effect of vitamin D supplementation on physical performance, exercise capacity, and daily physical activity in vitamin D-deficient, overweight non-western immigrants. A randomized double-blind, placebo-controlled trial was conducted to assess the effect of vitamin D on physical performance. A total of 130 participants were included. Eligibility criteria included overweight (BMI >27 kg/m^2^), 25-hydroxy vitamin D (25(OH)D) ≤50 nmol/l, and an age range of 20–65 years. The intervention group received 1200 IU vitamin D3 daily for 4 months; the control group received placebo. Both groups received 500 mg calcium daily. Outcome measures included physical performance (physical performance score), exercise capacity (a 6-min walk test (6-MWT)), and daily physical activity (questionnaire and accelerometer). There was no significant effect on physical performance, exercise capacity, or physical activity in the intention to treat analysis. In an explorative *post hoc* analysis restricted to participants reaching a serum 25(OH)D concentration of >60 nmol/l after intervention, there was an improvement of 19 m in the 6-MWT compared with the control group (*P*=0.053). Moderate dose vitamin D supplementation did not significantly improve physical performance, exercise capacity, or physical activity. However, when 25(OH)D concentrations reached >60 nmol/l after intervention, there was a borderline significant improvement in exercise capacity. Although the clinical relevance is not clear, this is a promising result, as all participants were overweight and did not improve their overall activity levels.

## Introduction

Physical inactivity has been identified as the fourth leading risk factor for global mortality, while overweight and obesity are in the fifth place (1). Both factors are more prevalent among non-western immigrants in The Netherlands, compared with the indigenous population. According to recent statistics, 72% of non-western immigrants do not comply with the Dutch physical activity guidelines (2), while 45% are overweight (BMI ≥25 kg/m^2^; http://statline.cbs.nl/, retrieved May 1, 2014).

In line with the other risk factors, the prevalence of vitamin D deficiency is much higher among non-western immigrants: 41.3% of the Turkish, 36.5% of the Moroccan, and 45.3% of the Surinam Creole adults have a vitamin D deficiency (serum 25(OH)D lower than 25 nmol/l), compared with 5.9% of the indigenous Dutch population (3). Vitamin D deficiency is associated with health problems including diabetes mellitus, the metabolic syndrome, cancer, pulmonary diseases, and significant myopathy (4, 5). Regarding myopathy, the knowledge about the effect of vitamin D on muscle morphology and functioning in elderly is increasing. Several studies in elderly report a positive relationship between vitamin D status and physical performance (6, 7, 8, 9) and evidence that vitamin D supplementation improves physical performance (10, 11, 12). However, other studies failed to show a significant effect of vitamin D supplementation on muscle strength (13, 14, 15). Knowledge about vitamin D and muscle function in a younger adult population is limited. Vitamin D supplementation increased muscle power in young Arabic women (16) and improved physical performance in a small trial in 40 healthy volunteers with hypovitaminosis D (17), but not all studies showed an effect (18).

The aim of this study was to assess the effect of vitamin D3 supplementation on physical performance, exercise capacity, and daily physical activity in vitamin D-deficient, overweight, non-western immigrants in The Netherlands, aged 20–65 years. In addition, the physical activity profile of the participants was assessed.

## Subjects and methods

### Participants

The study population consisted of non-western immigrants, both men and women, aged between 20 and 65 years, with a BMI above 27 kg/m^2^, impaired fasting glucose (IFG: 5.6–6.9 mmol/l) and/or impaired random glucose (IRG: 7.8–11.1 mmol/l), and vitamin D deficiency (serum 25(OH)D between 10 and 50 nmol/l). Participants who used vitamin D supplements containing more than 200 IU/day were excluded. Other exclusion criteria were: being pregnant or lactating, the intention to become pregnant within the study period, severe vitamin D deficiency (serum 25(OH)D <10 nmol/l), concurrent medication or serious physical impairment that might interfere with the interpretation of the data of the study, and serious mental impairment. The participants were recruited in the VU University Medical Center (VUmc), by general practitioners, in mosques, community centers, or at health markets. Potential participants underwent prescreening including medical history, anthropometric measurements, and blood sampling to assess blood glucose and serum 25(OH)D. Eligible participants were invited for the baseline visit.

The current study reports a secondary outcome of a clinical trial that primarily investigated the effect of vitamin D3 supplementation on insulin resistance and β-cell function (19). The study was approved by the Medical Ethics Committee of the VUmc. All participants gave written informed consent in their native language before commencement of the study. A total of 130 participants, 52 men and 78 women, were included in the analyses.

### Study design and intervention

Eligible participants were randomized in blocks of 20, stratified by sex. In each consecutive block, participants were allocated in a 1:1 ratio to receive three tablets of vitamin D3 of 400 IU (a total of 1200 IU) or three placebo tablets per day for a period of 4 months. All participants received one tablet of calcium carbonate containing 500 mg elemental calcium per day. The vitamin D3 and placebo tablets were manufactured by Vemedia Manufacturing BV (Diemen, The Netherlands). The participants visited the VUmc three times during the study period (baseline visit, visit after 2 months, and visit after 4 months). During the baseline visit, they were advised to maintain their own diet and physical activity profile. Anthropometric measurements, the 6-min walk test (6-MWT), and accelerometry were conducted at the baseline visit and after 4 months. The physical performance tests and the LASA Physical Activity Questionnaire (LAPAQ) were performed at all study visits. Pill compliance was monitored during the study period; adherence was defined as more than 80% intake of the prescribed pills. Data were unblinded in June 2012, after all participants completed the trial, all data were entered in the database, and all primary analyses were performed (19).

### Physical performance tests

Physical performance was assessed by several tests: the time needed to walk 3 m along a rope, turn 180°, and walk back, as fast as possible without running (walking test); the time needed to stand and sit down on a chair five times with arms folded across the chest (chair stand test); and the ability to perform a tandem stand for at least 20 s (tandem stand). The walking test is an indicator of coordination, proximal muscle strength, and balance. The chair stand test and tandem stand are indicators of proximal muscle strength and balance respectively. The time that participants required for the walking test and chair stand test was divided into tertiles. This resulted in the following scoring categories: unable (score 0), slowest tertile (score 1), intermediate tertile (score 2), and fastest tertile (score 3). The tandem stand was categorized as follows: unable (score 0), able to hold the position for 1–19 s (score 1.5), and able to hold the position for at least 20 s (score 3). The physical performance score was calculated by summing up the three individual test scores, with nine points reflecting excellent performance. Our scoring procedure is based on those of Wicherts *et al*. (8), adjusted to the performance of our study population.

### Exercise capacity

The participants performed a 6-MWT, a valid and reliable measure of submaximal exercise capacity (20, 21). The test was performed in a flat, straight indoor 30-m course. The total distance walked as fast as possible in 6 min was recorded.

### Physical activity

The LAPAQ was used to assess the physical activity in minutes per day. Frequency and duration of activities were asked for walking, bicycling, gardening, light and heavy household activities, and a maximum of two sports. The LAPAQ is a reliable and validated instrument for classifying the physical activity in older people (22).

The physical activity was also assessed objectively with an Actigraph accelerometer (Model 7164). These monitors are reliable and valid indicators of physical activity among adults (23). The participants were asked to wear the monitor for 4 days while they were awake, and to take it off for swimming or bathing. The data of the accelerometers were scored and interpreted using the MeterPlus Software (version 4.2.1, Santech I. Meterplus). Any block of ≥20 consecutive zero counts (20 min), indicating a time period of no movement, was considered as non-wearing time (24). A valid day was determined by the approach described by Catellier *et al*. (25). As 3–4 days of monitoring are preferable to assess habitual physical activity (26), participants who wore the accelerometer for <3 valid days were excluded. The raw ActiGraph counts were categorized using the Freedson *et al*. (27) cut-off points to determine the time spent in sedentary, light, moderate, hard, and very hard activity levels. These cut-off points for adults are widely accepted as accurate and valid (28). A mean activity score was calculated by dividing the total counts per day by the wearing time (counts per minute) (24). Then, we determined the percentage of participants i) meeting the criterion of 150 min of moderate-to-vigorous physical activity (MVPA) per week and ii) meeting the international physical activity recommendations of 30 min of MVPA on 5 days/week, in bouts of minimal 10 min (29).

### Biochemical analysis

Serum 25(OH)D was measured in EDTA plasma samples stored at −80 °C, by isotope dilution–online solid-phase extraction liquid chromatography–tandem mass spectrometry (ID–XLC–MS/MS). Samples were extracted and analyzed by XLC–MS/MS (a Symbiosis online SPE system (Spark Holland, Emmen, The Netherlands) coupled to a Quattro Premier XE tandem mass spectrometer (Waters Corp., Milford, MA, USA)). The interassay CV was 9% at 25 nmol/l and 6% at 63 nmol/l (19).

### Statistical analysis

Differences in baseline characteristics between treatment and control groups were tested using independent *t*-tests for normally distributed continuous variables, the Mann–Whitney *U*-test for skewed distributed variables, and the Pearson *χ* ^2^ test for categorical variables. The Pearson correlation coefficients were calculated to assess baseline correlations. Skewed variables were log transformed.

Intention-to-treat analyses were performed for each follow-up moment using linear mixed models (LMMs). The LMM adjusts for dependencies between repeated responses and allows for missing data without introducing bias. Fixed effects were the factors i) time (with levels 0 (baseline), 2 months (if appropriate), and 4 months), ii) group (with levels intervention and placebo) and iii) the group by time interaction. An unstructured covariance matrix was chosen for the repeated measures. The effect of interest was the group by time interaction, which can be interpreted as the difference score (follow-up – baseline) for the intervention group compared with the control group during the follow-up period. This effect was named the estimated effect (Table 3). All effects were adjusted for sex, age, BMI, and baseline 25(OH)D. Then, per protocol analyses were performed, including i) all participants with baseline serum 25(OH)D values below 25 nmol/l (*n*=74), ii) participants with compliance above 80% (*n*=81), and iii) participants with 25(OH)D above 60 nmol/l after treatment (intervention group only; vitamin D group: *n*=27; and control group: *n*=55). In all the per protocol analyses, five participants were excluded: three had a BMI <25 kg/m^2^ at baseline, one had type 2 diabetes at baseline, and one violated the protocol due to holiday and sun exposure. In case of abnormal distributed residuals, the variables were log or square-root transformed for the LMM analyses. Statistical significance for all analyses was set at *P*<0.05. The analyses were performed using SPSS 15.0 (version 15.0.1, SPSS, Inc.).

## Results

### Participants and baseline characteristics

A total of 130 participants, 52 men and 78 women, were included in the analyses. Of the persons who did not complete the study, 11 withdrew consent, three did not adhere to the protocol due to long stay in a foreign country, four started antidiabetic medication, one started u.v. light therapy, and one became pregnant (resulting *n*=110). In the physical performance analyses, we excluded two additional participants: one with missing data and the other who did not understand the test procedures resulting *n*=108. Owing to physical complaints (joint pain, palpitations, dyspnea, and nausea), six participants did not complete the 6-MWT (resulting *n*=104). Regarding accelerometry, due to malfunction of the devices (22 participants), insufficient valid measurement days (20 participants), holidays (two participants), and one lost device, 45 participants were excluded from the analyses (resulting *n*=65).

**Figure 1:**
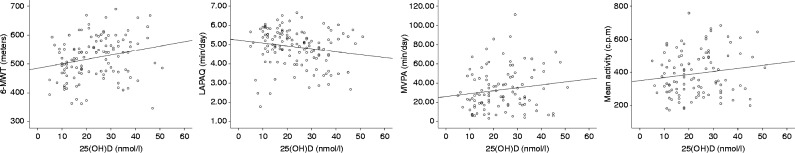
Scatterplots of the association between baseline 25(OH)D and the score on the 6-min walk test (6-MWT), minutes of physical activity per day measured by the LASA Physical Activity Questionnaire (LAPAQ), minutes of moderate-to-vigorous physical activity (MVPA) per day, and the mean activity score (counts per minute). The lines depicted are the linear regression lines.

There were no significant baseline differences between intervention and control groups (Table 1). Baseline serum 25(OH)D was significantly (*P*=0.012) associated with the baseline distance walked during the 6-MWT, but not with other physical function tests (Table 2 and Fig. 1).

### Change in 25(OH)D and PTH after intervention

The vitamin D supplements were well tolerated. Five participants discontinued calcium because of constipation and other gastrointestinal complaints. They were advised to optimize their dairy intake.

Overall pill adherence was 83% for the vitamin D supplements and 82% for calcium and no differences between intervention and control groups were found.

After therapy, mean 25(OH)D concentrations increased from 25 nmol/l (s.d. 11) to 58 nmol/l (s.d. 12) after 2 months and to 60 nmol/l (s.d. 16) after 4 months in the intervention group. In the control group, 25(OH)D concentrations remained stable, 22 nmol/l (s.d. 11) at baseline, 24 nmol/l (s.d. 16) after 2 months, and 23 nmol/l (s.d. 15) after 4 months. There was a significant difference between intervention and control groups after 4 months of 38 nmol/l (95% CI 32; 44, *P*<0.001).

At baseline, 49% of the participants in the intervention group had a 25(OH)D value <25 nmol/l and 51% had a value >25 nmol/l. After 2 months of treatment, 100% of the participants in the intervention group had a 25(OH)D value >25 nmol/l, 76.4% had a value >50 nmol/l, and 9.1% had a value >75 nmol/l. The percentages after 4 months were 98.1, 79.2, and 15.1% respectively.

The PTH concentrations decreased in the intervention group during the treatment period. After 4 months, there was a significant change in PTH concentrations between treatment and control groups (*P*=0.03). Median serum PTH after 4 months was 7 pmol/l (interquartile range (IQR), 6–9) in the treatment group and 8 pmol/l (IQR 6–11) in the control group.

### Effect of the intervention on physical performance and activity

The intention-to-treat model-based means, treatment effects, and 95% CI for the outcome measures by performing LMMs are shown in Table 3. No significant differences between intervention and control groups were observed with regard to the physical performance score, the 6-min walk distance, physical activity measured by the LAPAQ, and accelerometry. The per protocol analyses revealed that the difference in 6-min walk distance between intervention and control groups was borderline statistically significant when only the participants reaching a serum 25(OH)D concentration of >60 nmol/l after intervention (vitamin D group, *n*=27 and controls, *n*=63) were selected (per protocol analysis 3). This resulted in a treatment fixed effect of 19 (95% CI 0.2; 38, *P*=0.053, indicating that the improvement in the 6-MWT was 19 m more in the intervention group than in the control group. The other per protocol analyses revealed no relevant differences (data not shown).

### Physical activity profile

Regarding physical activity recommendations, 67% of participants in the intervention group and 67% of participants in the control group met the criteria of 150 min of MVPA per week. The values were, respectively, 4.4 and 8.8%, when the MVPA was performed in bouts of minimal 10 min (Table 1).

## Discussion

In this trial, vitamin D supplementation did not significantly improve physical performance, exercise capacity, or daily physical activity in vitamin D-deficient, overweight, and non-western immigrants. However, an explorative analysis selecting participants with a 25(OH)D concentration >60 nmol/l after intervention showed a borderline significant improvement in exercise capacity. In addition, in this study, the percentage of participants meeting international physical activity guidelines was extremely low (<10%).

Current research assessing the effect of vitamin D supplementation on physical performance in older persons, the most studied population, yields controversial results. Various issues may explain why some trials did not find any effect of supplementation, including a relatively small increase in serum 25(OH)D (13, 14), a normal vitamin D status at baseline (14), exceptionally good physical function (14), and the confounding effects of training (15). In a trial by Wicherts *et al*. there were no between-group or within-group differences in the chair stand test and handgrip strength after 6 months of vitamin D supplementation in participants with a mean age of 41.3 years. In this study, however, vitamin D supplementation was compared with sunshine exposure advice and there was no control group (30). On the other hand, Gupta *et al*. reported a significant enhancement of muscle strength and a gain of 16 m on the 6-MWT after 6 months supplementation with vitamin D3 and calcium in a study population aged 20–40 years. Despite their smaller study population, they had less variation in the test scores in the 6-MWT. Furthermore, they gave a higher dose of vitamin D (60 000 IU D3/week for 8 weeks followed by 60 000 IU/month for 4 months), resulting in higher serum 25(OH)D values after 2 months (17). Another study with younger participants did not find any effect of vitamin D3 supplementation of 1000 IU or 400 IU on muscle strength or power in non-western immigrants in Norway. In this trial, not all participants were vitamin D deficient at baseline and only 57% in the 1000 IU supplementation group and 38% in the 400 IU group reached 25(OH)D concentrations ≥50 nmol/l (18). In our study, most participants (79%) reached a 25(OH)D concentration of >50 nmol/l after 4 months of supplementation of 1200 IU vitamin D and 15% reached a concentration of >75 nmol/l. Some studies suggest higher 25(OH)D serum concentrations. Diamond *et al*. (31) concluded that 5000 IU vitamin D3 daily might be more effective than 2000 IU in achieving optimal serum 25(OH)D concentrations in vitamin D-deficient subjects. Then, Visser *et al*. (6) reported that 25(OH)D concentrations should be as high as 75 nmol/l to avoid loss of grip strength. The significant baseline correlation between the plasma 25(OH)D concentrations and the performance on the 6-MWT in addition to the borderline significant improvement of meters walked when 25(OH)D concentrations were >60 nmol/l after intervention may support this suggestion and warrant further studies.

A reduction of complaints such as fatigue might be the reason why our subjects in the intervention group, although not significantly, improved their walking distance in the 6-MWT. Treating hypovitaminosis D reduced subjective complaints of muscle discomfort within 1–1½ months in a Danish study (16). Furthermore, vitamin D supplementation seems to improve muscle integrity in vitamin D-deficient patients. The presence of the vitamin D receptor (VDR) has been detected in mice by various techniques (32, 33, 34). Bischoff *et al*. (35) were the first to report the detection of the VDR in human muscle cells. A recent study, however, did not find VDR expression in skeletal muscle (36). Several reasons can explain this discrepancy: the possibility of tight protein binding of the VDR to DNA, differences in experimental conditions, and differences in VDR expression throughout the various stages of muscle differentiation (37). A recent pilot study showed that vitamin D supplementation increased VDR concentration in muscle fiber and muscle fiber size in older women (38). This study is in line with the work by Sato *et al*. (39), which demonstrated that vitamin D supplementation increased the mean diameter of type 2 fibers in post-stroke vitamin D-deficient patients. Still, further assessment of the effects of vitamin D on muscle function is desirable.

This study has several strengths. First, we evaluated the effect of vitamin D supplementation in a double-blind and placebo-controlled manner. Secondly, only participants with a vitamin D deficiency were included and the participants had very low activity levels. Finally, we measured the physical activity completely by both a comprehensive questionnaire and, more objectively, accelerometry. This provided exclusive information about the intensity of the physical activity of a population prone to health problems.

This study has some limitations. Regarding recent insights, our supplementation dose of 1200 IU vitamin D3/ day was low. Currently, a supplementation dose of 2000 IU daily is common in trials with vitamin D-deficient patients. Furthermore, overweight patients may require higher supplementation doses, as the increase in plasma 25(OH) is lower when the BMI is more than 25 kg/m^2^ (30). It should be noted, however, that high doses of vitamin D may be hazardous, as was illustrated by an Australian trial of older community-dwelling women who received a yearly vitamin D supplement of 500 000 IU or placebo. In that study, an increase in falls and fractures was observed in the vitamin D group, mainly during the first 3 months after the high vitamin D dose was given (40). Regarding muscle strength, the supplementation period of our trial could have been longer. This is implied by the finding of a Japanese group that it may take 6–12 months for histological changes in muscle fibers to recover with vitamin D supplementation (39). At last, as our trial primarily investigated the effect of vitamin D supplementation on insulin resistance and β-cell function, the power analysis for the study was based on insulin resistance parameters. However, several other trials investigating the effect of vitamin D supplementation on physical performance had a lower or comparable number of subjects (13, 14, 15, 17).

This study provides useful information about the activity profile of overweight, non-western immigrants. The consequences of physical inactivity are tremendous. It is estimated to be the principal cause for ∼30% of ischemic heart disease, 27% of diabetes, and 21–25% of breast and colon cancer burden (1). As <10% of the participants of our study met the criteria of international physical activity guidelines, a lifestyle intervention could be essential for the health of overweight non-western immigrants.

When 25(OH)D concentrations were >60 nmol/l after intervention, there was borderline improvement of physical exercise capacity in non-western immigrants. In older patients with heart failure, the minimum clinically important change for the 6-MWT was 30 m (41). The clinical relevance of our finding, an improvement of 19 m in overweight adults, is yet unclear. However, it is a promising result, as all participants were overweight and did not improve their overall activity levels. This positive effect might be the result of improving muscle integrity, decreasing fatigue, and other symptoms through vitamin D supplementation. Hence, correcting hypovitaminosis D might be essential in this population to optimize the conditions for other health interventions. Further research, however, is required to determine whether vitamin D supplementation could improve physical performance and exercise capacity.

## Author contribution statement

The study was designed by E M W Eekhoff, P Lips, N M Van Schoor, M M Oosterwerff, M N M Van Poppeland conducted by M M Oosterwerff, E M W Eekhoff, R Meijnen, M N M Van Poppel and P Lips. M H H Kramer assisted in patient recruitment. M M Oosterwerff, R Meijnen, N M Van Schoor, D L Knol and E M W Eekhoff performed data analysis. The paper was written by M M Oosterwerff, R Meijnen, N M Van Schoor, M N M Van Poppel, P Lips and E M W Eekhoff. All authors read the paper and approved the final version.

## Figures and Tables

**Table 1:** Baseline characteristics of the intervention and control groups. Data are expressed as means±s.d. or median (interquartile range).

	**Intervention** (*n*=65)	**Control** (*n*=65)	***P***
Age (years)	48.9±10.3	51.5±10.5	0.16
Sex (% men)	40	40	1.00
BMI (kg/m^2^)	32.1±4.8	33.2±5.1	0.25
Waist-to-hip ratio	0.90±0.08	0.92±0.07	0.17
Systolic BP (mmHg)	133±16	135±16	0.43
Diastolic BP (mmHg)	80±12	80±12	0.93
25(OH)D (nmol/l)	25.0±10.7	21.7±10.5	0.08
PTH (pmol/l)	8.4 (6.6–11.2)	9.5 (6.4–12.8)	0.54
Ethnicity			0.34
Moroccan (%)	40	37	
Surinam (%)	19	32	
Turkey (%)	19	20	
Others (%)	23	11	
Physical performance (0–9)	6.78±1.89	7.00±1.50	0.47
Chair stand (0–3)	1.95±0.97	2.06±0.85	0.54
Tandem stand (0–3)	2.81±0.57	2.95±0.26	0.08
Walking test (0–3)	2.02±0.83	2.00±0.78	0.91
6-MWT (m)	525±72	517±73	0.53
LAPAQ (min/day)	140 (66–271)	165 (101–258)	0.46
MVPA (min/day)	26 (18–44)	30 (16–49)	0.79
Mean activity (c.p.m.)	389±128	394±140	0.83
% 150 min MVPA/week	67	67	1.00
% 150 min MVPA/week^a^	4.4	8.8	0.46

6-MWT, 6-min walk test; LAPAQ, LASA Physical Activity Questionnaire; MVPA, moderate-to-vigorous physical activity; c.p.m., counts per minute. *P* values of independent *t*-tests for continuous variables and the Mann–Whitney *U* tests for skewed variables; differences in frequencies by the Pearson *χ* ^2^ tests.

a In bouts of 10 min.

**Table 2:** Baseline correlations between 25(OH)D and physical performance and activity parameters.

	***R***	***P***
Physical performance (0–9)^a^	0.03	0.73
6-MWT (m)	0.23	0.01
LAPAQ (min/day)^a^	−0.17	0.06
MVPA (min/day)	0.15	0.13
Mean activity (c.p.m.)	0.15	0.15

6-MWT, 6-min walk test; LAPAQ, LASA Physical Activity Questionnaire; MVPA, moderate-to-vigorous physical activity; c.p.m., counts per minute.

a Log transformed.

**Table 3:** Linear mixed model-based means at baseline and follow-up, and treatment effects (95% CI).

	**Intervention**	**Control**	**Effect (95% CI)** ^a^	***P***
Physical performance				
Model 1				
Baseline	6.8	7.0		
2 months	7.1	7.0	0.3 (−0.2; 0.8)	0.27
4 months	7.2	7.2	0.3 (−0.1; 0.7)	0.18
Overall effect: *F*(2, 107)=1.03, *P*=0.36				
Model 2				
Baseline	6.7	7.2		
2 months	7.0	7.2	0.3 (−0.2; 0.8)	0.24
4 months	7.2	7.4	0.3 (−0.1; 0.8)	0.13
Overall effect: *F*(2, 107)=1.30, *P*=0.28				
**6-MWT**				
Model 1				
Baseline	525	517		
4 months	538	522	7 (−9; 24)	0.38
Model 2				
Baseline	523	529		
4 months	537	535	8 (−8; 24)	0.33
**LAPAQ** ^b^				
Model 1				
Baseline	12	13		
2 months	13	13	1 (−0.1; 3)	0.10
4 months	12	13	0 (−1; 1)	0.99
Overall effect: *F*(2, 108)=1.71, *P*=0.19				
Model 2				
Baseline	12	13		
2 months	13	12	1 (−0.2; 3)	0.10
4 months	11	12	0 (−1; 1)	0.96
Overall effect: *F*(2, 110)=1.83, *P*=0.17				
**MVPA**				
Model 1				
Baseline	32.1	33.3		
4 months	31.7	35.7	−3.9 (−14.2; 6.5)	0.46
Model 2				
Baseline	32.9	34.3		
4 months	31.6	37.0	−3.9 (−14.3; 6.5)	0.46
Mean activity				
Model 1				
Baseline	389	394		
4 months	375	426	−44 (−123; 35)	0.27
Model 2				
Baseline	387	398		
4 months	373	430	−46 (−125; 33)	0.25

Model 1: univariable; Model 2: adjusted for sex, age, baseline BMI, and baseline 25(OH)D. Covariates were fixed at the mean values; 6-MWT, 6-min walk test; LAPAQ, LASA Physical Activity Questionnaire, MVPA, moderate-to-vigorous physical activity; c.p.m., counts per minute.

a The estimated effect is calculated as (the model-based mean after follow-up−the model-based mean at baseline for the intervention group)−(the model-based mean after follow-up−the model-based mean at baseline for the control group). For example, the estimated effect for LAPAQ, follow-up of 2 months: (13–12)−(13–13)=1.

b Square-root-transformed variables.
